# Drastic Response to Olaparib in a Patient With Metastatic Castration‐Resistant Prostate Cancer Harboring BRCA2 Alterations and Near‐Threshold Tumor Mutational Burden

**DOI:** 10.1002/iju5.70144

**Published:** 2026-01-28

**Authors:** Kotaro Yokota, Takeo Kosaka, Tatsuaki Daimon, Shinnosuke Fujiwara, Kohei Nakamura, Hiroshi Nishihara, Mototsugu Oya

**Affiliations:** ^1^ Department of Urology Keio University School of Medicine Tokyo Japan; ^2^ Department of Urology Eiju General Hospital Tokyo Japan; ^3^ Department of Urology Nerima General Hospital Tokyo Japan; ^4^ Keio Cancer Center Keio University School of Medicine Tokyo Japan

**Keywords:** BRCA mutation, near‐threshold TMB, prostate cancer, TMB‐H

## Abstract

**Introduction:**

BRCA2 alterations and high tumor mutational burden (TMB‐H) are responsible for prostate cancer; however, their co‐occurrence is uncommon, and evidence for PARP inhibition in the castration‐sensitive setting remains limited. We describe a case of metastatic castration‐resistant prostate cancer (CRPC) harboring both biomarkers, showing a marked response to olaparib.

**Case Presentation:**

A 74‐year‐old man presented with urinary retention. Initial prostate‐specific antigen (PSA) level was 11 ng/mL. Follow‐up MRI revealed bilateral PI‐RADS 5 lesions with seminal‐vesicle invasion. Biopsy confirmed adenocarcinoma (Gleason score 5 + 5 = 10). Staging revealed osseous and 30‐mm right internal iliac nodal metastasis. Genomic profiling identified a pathogenic BRCA2 mutation and near‐threshold TMB. Chemohormonal therapy was discontinued early owing to severe infection, and olaparib was initiated. Over 3 months, MRI showed further regression of the primary lesion and nodal disease, and PSA and SCC decreased.

**Conclusion:**

In metastatic CRPC harboring a BRCA2 mutation and near‐threshold TMB, olaparib produced clear radiological and serological responses.

## Keynote Message

In this report, we describe a case of metastatic castration‐resistant prostate cancer (mCRPC) with both BRCA2 alteration and near‐threshold tumor mutational burden, in which the PARP inhibitor olaparib showed a remarkable response. The coexistence of BRCA2 alteration and TMB‐H is rare in prostate cancer, but in this case, their presence was related to treatment selection. We present this case in the hope that it will serve as a reference for therapeutic decision‐making in similar patients.

## 1 Introduction

Prostate cancer is the second most commonly diagnosed malignancy among men worldwide [1]. Its risk factors include age, ethnicity, as well as germline and somatic gene alterations [2]. As such, the 2023 Japanese Guidelines for Prostate Cancer recommend genomic testing to facilitate the use of companion diagnostics [3]. Based on the PROfound trial, the poly(ADP‐ribose) polymerase (PARP) inhibitor olaparib is recommended for castration‐resistant prostate cancer (CRPC) harboring pathogenic BRCA1/2 alterations [4], which occur in approximately 12% of CRPC cases [5]. For tumors with a high tumor mutational burden (TMB‐H), the KEYNOTE‐158 study demonstrated the tumor‐agnostic efficacy of the programmed death‐1 (PD‐1) inhibitor pembrolizumab, leading to tissue‐agnostic approval [6, 7]. However, TMB‐H is rare in patients with prostate cancer (approximately ≈1.5%) [8]. Although BRCA alterations are generally rare in TMB‐H tumors across most cancer types, they may be relatively common in TMB‐H prostate cancer [9].

As PROfound enrolled only patients with CRPC [4], the efficacy of olaparib in BRCA1/2‐mutated CSPC, as well as the optimal sequencing of olaparib and pembrolizumab in tumors harboring both BRCA alterations and TMB‐H, remains unclear. Here, we report a rare case of metastatic CRPC with a pathogenic BRCA2 mutation and near‐threshold TMB that demonstrated a marked radiological and serological response to olaparib.

## 2 Case Presentation

A 74‐year‐old man presented with urinary retention following a herpes zoster infection. His medical history included thyroid‐associated orbitopathy, chronic kidney disease, and hypertension. At the initial visit in July of 2023, the prostate‐specific antigen (PSA) level was 11 ng/mL. Pelvic magnetic resonance imaging (MRI) revealed no definite malignant findings. Serum tumor markers were squamous cell carcinoma antigen (SCC) 9.4 ng/mL and neuron‐specific enolase (NSE) 9.9 ng/mL. Although catheterization relieved the retention, the PSA level declined to only 6 ng/mL. Five months later, follow‐up MRI revealed PI‐RADS 5 lesions in the bilateral peripheral zones with seminal‐vesicle invasion.

Transrectal biopsy demonstrated adenocarcinoma with a Gleason score of 5 + 5 = 10, all 12 cores were positive, with right seminal‐vesicle involvement. Contrast‐enhanced computed tomography (CT) indicated a 30‐mm metastasis in the right internal iliac lymph node, and bone scintigraphy revealed metastases in the right sacrum and left ischium. The patient's clinical stage was cT3b, N1, M1b. Pretreatment laboratory tests reported PSA 8.95 ng/mL and SCC 22.5 ng/mL.

Given the high risk of metastatic CSPC, triplet therapy with darolutamide, docetaxel, and the gonadotropin‐releasing hormone (GnRH) antagonist degarelix was initiated. A next‐generation sequencing (NGS) panel was submitted simultaneously. After one docetaxel cycle, the metastatic lymph node had shrank to 13 mm, and the SCC antigen level decreased to 5.2 ng/mL. However, after two cycles under chemotherapy‐induced immunosuppression, the patient developed a lung abscess that progressed to septic shock. Chest tube drainage and 5 weeks of broad‐spectrum antibiotics were required, and continuation of triplet therapy was deemed infeasible.

**FIGURE 1:**
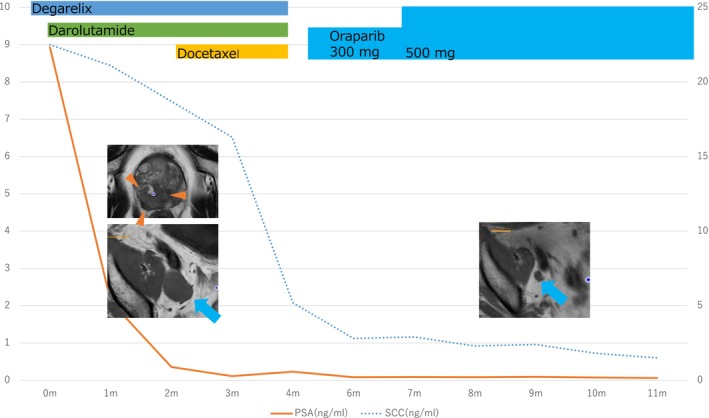
Treatment course, serum markers, and imaging findings. Timeline of systemic therapy indicating administration of degarelix, darolutamide, docetaxel, and olaparib. Time course of serum PSA and SCC. Baseline and on‐treatment CT images. Triangles denote the primary tumor; arrows indicate lymph‐node metastases.

NGS identified a pathogenic BRCA2 mutation and near‐threshold TMB (9.6 mutations/megabase) (Table 1). Considering both treatment toxicity and expected efficacy, olaparib was initiated 16 months later. By 21 months, MRI showed further shrinkage of the primary prostatic lesion and metastatic lymph nodes, and the SCC level had declined to 1.5 ng/mL (Figure 1).

**TABLE 1:** Genomic findings from FoundationOne CDx (BRCA2 alteration and TMB).

FundationOne CDx	
BRCA2	Loss
Tumor mutational burden (TMB)	High
Microstatellite status	MS‐stable

## 3 Discussion

BRCA1 and BRCA2 encode critical proteins involved in homologous‐recombination repair [10]. Pathogenic variants in these genes markedly increase lifetime cancer risk; for prostate cancer, the relative risk increases by up to 3.8‐fold with BRCA1 and up to 8.6‐fold with BRCA2 [11]. The introduction of PARP inhibition has improved the historically poor prognosis of BRCA‐mutated prostate cancer, and olaparib is now the standard of care for BRCA‐mutated castration‐resistant disease [4, 12]. Contrarily, pembrolizumab—approved for tumors with TMB‐H lacks prostate‐specific evidence, and an optimal strategy for tumors harboring both BRCA mutations and TMB‐H has yet to be established [7].

In the present case, continuation of cytotoxic chemotherapy was precluded by severe infection. Genomic profiling revealed both a pathogenic BRCA2 mutation and TMB‐H, expanding therapeutic options. Based on the stronger foundational evidence, olaparib was prioritized and achieved remarkable regression of both the primary lesion and metastatic sites. Although the PSA level was low, the tumor progressed rapidly—an evolution often associated with androgen‐receptor independence or neuroendocrine differentiation—suggesting that a DNA‐repair–targeted approach was biologically appropriate [13, 14].

In this case, aggressive behavior was observed, with new nodal and osseous metastases emerging within 5 months. Accordingly, the referring hospital measured SCC and carcinoembryonic antigen levels to exclude alternative histology. SCC was elevated; however, systemic imaging revealed no other primary tumor, and retrospective immunohistochemistry for SCC in the biopsy specimen was negative. The mechanism by which SCC acted as a marker of disease activity therefore remains unclear. Sampling error, whereby an SCC‐positive focus is missed, is a plausible explanation. Additional studies are required to clarify the relationship between the SCC kinetics and BRCA‐positive prostate cancer.

Should olaparib eventually lose efficacy in this patient, treatment with pembrolizumab is planned.

## Ethics Statement

Yes (IRB approval nos.: 2016008, 20200046).

## Consent

Yes (including written consent for publication).

## Conflicts of Interest

The authors declare no conflicts of interest.

## Data Availability

Data sharing not applicable to this article as no datasets were generated or analyzed during the current study.
